# Body Composition and Fitness Characteristics of Firefighters Participating in a Health and Wellness Program: Relationships and Descriptive Data

**DOI:** 10.3390/ijerph192315758

**Published:** 2022-11-26

**Authors:** Robert G. Lockie, Joseph M. Dulla, Daniel Higuera, Kristina A. Ross, Robin M. Orr, J. Jay Dawes, Tomas J. Ruvalcaba

**Affiliations:** 1 Department of Kinesiology, California State University, Fullerton, CA 92831, USA; 2 Tactical Research Unit, Bond University, Robina, QLD 4229, Australia; 3 Fire Technology Department, Santa Ana College, Santa Ana, CA 92706, USA; 4 Human and Sport Performance, Rocky Mountain University, Provo, UT 84606, USA; 5 Tactical Fitness and Nutrition Lab, Oklahoma State University, Stillwater, OK 74078, USA

**Keywords:** body composition, cardiovascular disease, first responders, flexibility, maximal aerobic capacity, muscular endurance, muscular strength, tactical, waist circumference, waist-to-hip ratio

## Abstract

This study investigated body composition and fitness test relationships from firefighters participating in a health and wellness program and categorized firefighters according to population norms relative to sex and age. Data from 270 firefighters (men = 258, women = 12) were analyzed, including body composition (body mass index [BMI], body fat percentage [BF%], waist circumference [WC], waist-to-hip ratio) and fitness (sit-and-reach, grip strength, leg press, crunches, push-ups, maximal aerobic capacity [V̇O_2max_]) tests. Mann–Whitney U-test analysis (*p* < 0.05) showed that male firefighters had a greater WC, WHR, grip strength and leg press. Female firefighters had a greater BF% and better sit-and-reach. Partial correlations controlling for sex indicated 22/24 correlations between body composition and fitness were significant (*r* = −0.143–−0.640). ~52% of firefighters were overweight, and 25% were Obesity Class I-III. ~76% had an increased risk of cardiovascular disease (CVD) considering BMI and WC. ~22% were fatter than average-to-overfat considering BF%. Most firefighters (73–94%) were good-to-excellent in sit-and-reach, grip strength, and push-ups; average-to-well above average in crunches; average-to-above average in leg press; and had good-to-superior V̇O_2max_. Although most firefighters had better fitness compared to the general population, many had increased CVD risk. The data highlighted the need for comprehensive approaches to improving firefighter health and decreasing CVD risk.

## 1. Introduction

The work environments for firefighters can involve extreme conditions, where temperatures may exceed 500 °C [1], presenting a high risk of thermal injury [2]. Firefighters are often exposed to toxic environments and smoke inhalation [2,3,4], and will wear personal protective equipment and use a self-contained breathing apparatus in an attempt to limit exposure. While necessary, this protective equipment can equate to an additional load of greater than 20 kg [1], which increases the stress and difficulty (e.g., increased aerobic and anaerobic energy cost, decreased mobility, increased perception of effort) of fireground job tasks [5,6]. Indeed, firefighters are required to perform numerous demanding tasks when on the fireground, including operating hose lines, carrying equipment, forcible entries, ladder raises, crawling and searching, and victim or casualty drags [7,8,9]. Consequently, fitness can be an important contributor to a firefighter’s job performance. Numerous studies have indicated the value of aerobic fitness for firefighters [10,11,12], with a minimum maximal aerobic capacity (V̇O_2max_) of 45 milliliters per kilogram per minute (ml/kg/min) recommended for applicants to a firefighting academy [8]. Further, Rhea et al. [7] documented that upper-body strength (measured by a five-repetition maximum bench press and hand grip) significantly (*p* ≤ 0.05) correlated with completion time for a 65.6 m hose pull, 22 kg hose pack stair climb, 30 m 80 kg victim drag, and 30.3 m equipment hoist (equipment load = 16 kg) in incumbent firefighters.

In addition to the physical job demands, another consideration for firefighters is risk of cardiovascular disease (CVD) [13,14,15,16]. As noted, firefighters often work in hot, smoky, and toxic environments, and exposure to heat, smoke, and toxins will increase cardiovascular strain [17,18]. There are also lifestyle factors that contribute to CVD risk in firefighters. This includes low physical fitness, a sedentary lifestyle, obesity, cigarette smoking, and hypertension [14,15,16]. Even with the recognized importance of fitness relative to job performance [7,8,10,11,12], the fitness of firefighters tends to decline once they leave their respective training academy and start working at a fire station [19]. Previous research has also shown that the aerobic fitness of firefighters tends to decline over their career [20]. For older firefighters, a further issue is a change in job roles, where they may transition into more supervisory positions [21]. As noted by Dobson et al. [21], this can lead to a decline in physical activity and an increase in sedentary behavior (e.g., more office work).

As a result of the job demands and existence of many firefighters with less-than-optimal health and fitness profiles [14], fire departments often use health and wellness programs to encourage improved health outcomes for their personnel. These types of programs for firefighters (and first responders in general) are typically multi-faceted with different emphases to cater to the diverse population of personnel [22]. Some of the interventions incorporated into health and wellness programs for first responders include: body composition and fitness testing, strength and conditioning programs, body weight management, nutrition, injury prevention, chronic disease prevention, alcohol and drug use interventions, stress management, and trauma resilience [22,23,24,25]. Health and wellness programs are typically voluntary and incentivized (3), so not all personnel may participate. However, if these programs could contribute to enhanced health and wellness profiles for personnel, this may lead to better job performance, career longevity, and the personal life outcomes for individual firefighters.

Despite the potential importance of health and wellness programs for firefighters, there is relatively little research that details their effectiveness [23,26]. There has been some analysis of health and wellness programs for police officers [22,24]. For example, Lockie et al. [24] found few significant differences in fitness (e.g., sit-and-reach, grip strength, vertical jump, sit-ups, push-ups, V̇O_2max_) across 3 years of a health and wellness program for police officers. The absence of fitness declines within the overall sample of officers was noted as a positive outcome by Lockie et al. [24]. This was because similar to firefighters [19,20], fitness tends to decline over the course of a police officer’s career [27,28,29]. Nevertheless, cross-sectional analyses of firefighter [20] and police officer [24] health and wellness programs have indicated that even with program participation, physical fitness may decline with increased age in individual personnel. Specific to police officers, however, Lockie et al. [24] observed that it was a positive outcome that older personnel were participating within the program. 

More scientific analysis is required on health and wellness programs for firefighters [23,26]. For instance, obesity is a contributing factor to CVD risk [16,30,31], and firefighters as a group can have a high prevalence of high body fat [15,21,32,33]. Detailing the relationships between body composition and fitness in firefighters participating in a health and wellness program is noteworthy. This is because these relationships could highlight approaches that could be adopted within a health and wellness program to benefit firefighter health and fitness (i.e., methods to decrease body fat that could be reflected in fitness test performance). Moreover, documenting whether firefighters display better health and fitness relative to the general population could provide some evidence of program effectiveness. The provision of descriptive health and fitness data for firefighters from a health and wellness program could document what characteristics could be targeted by specific interventions in this population.

Therefore, this study analyzed archival data collected from one fire department by staff working for a health and wellness program to achieve several goals. The data included measures of body composition (body mass index [BMI], body fat percentage [BF%], waist circumference [WC], and waist-to-hip ratio [WHR]) and fitness (sit-and-reach, combined grip strength from the right and left hands, one-repetition maximum [1 RM] leg press, abdominal crunches, push-ups, and estimated maximal aerobic capacity [V̇O_2max_]). Firstly, this study compared the data from the male and female firefighters to ascertain any between-sex differences. It was important to analyze the sexes separately as it is necessary to profile female firefighters [34] given that many departments are actively trying to recruit more women [35]. Secondly, partial correlations controlling for sex were used to analyze the relationships between the body composition and fitness test. Previous research has shown some relationships between measures of body fat and fitness in other first responder populations [36,37,38,39]. However, in police officers Lockie et al. [22] has stated that measures of health and fitness are relatively disparate, so it was important to document whether this was also the case for firefighters. Lastly, and most notably, this study provided a descriptive analysis of the health and fitness of firefighters who were participating in a health and wellness program by comparing their results to established normative data from the general population [40,41]. It is critical to identify whether firefighters are actually healthy and fit individuals relative to the general population [42]. This is because firefighters who are not as healthy and fit may negatively impact their own safety or that of their colleagues and the communities in which they serve. It was hypothesized that the male firefighters would outperform the female firefighters in the fitness tests. It was also hypothesized that there would be significant relationships between body composition and fitness in the firefighters, but the strength pf the relationships would be small. It was lastly hypothesized that the majority of firefighters participating in the health and wellness program would demonstrate better health and fitness relative to general population norms.

## 2. Materials and Methods

### 2.1. Subjects

Anonymized and deidentified archival data from structural incumbent firefighters from one city fire department who participated in a health and wellness program administered by a community college program were utilized for this study. This study included 270 firefighters, comprised of 258 males and 12 females. Involvement within the program was voluntary. However, financial incentives were awarded to individuals who were able to maintain or improve their fitness levels to at or above the 60th percentile of internal occupational norms using age, sex, and department benchmarks. The occupational norms were developed from previous data collected on paid career structural incumbent firefighters that have completed the fitness tests included in the health and wellness program (these norms did not factor into the current investigation). The fire department from this study employs approximately 400 personnel [43], so the sample was equivalent to about 68% of the workforce. Inclusion criteria were that firefighters had to be a member of the fire department and capable of performing and completing all the health and fitness testing protocols (and thus, had full available datasets). Firefighters who could not complete the program’s protocol in its entirety (and thus had incomplete datasets), were omitted. Based on the archival nature of this analysis, the institutional ethics committee approved the use of pre-existing data (HSR-20-21-58). The study followed the recommendations of the Declaration of Helsinki [44].

### 2.2. Procedures

Data collection was conducted at the fire station over the course of two days as part of the department’s voluntary annual wellness and fitness testing. The testing battery has been adopted for many years as part of the community college program, such that staff have built a large bank of historical data. The information derived from this testing has been internally to track firefighter fitness and provide information to individual fire departments, which is part of the reason why the tests were adopted in the context of this research. Firefighters were assigned different orientation and testing days, either during occupational hours or on off days. The first day involved firefighters registering for the health and wellness program and completing all the necessary paperwork (medical release form, fitness assessment disclaimer, and health history questionnaire). The second day included measurement of body composition and fitness. Firstly, firefighters completed an informed consent form and pre-test checklist administered by the program staff. Following this, all the health and physical fitness assessments were conducted, which were standard within the program. The tests were broken down into two categories (body composition and fitness) and were completed in the order described hereafter. Prior to the fitness tests, the following warm-up was completed: 3–5 min on a stationary bicycle, followed by a 7–10 min movement preparation with each exercise being held for 15 s (straight arms behind the back, chicken wing, arms straight up above the head, forward lunge, trunk twist, supine hip and knee flexion, sitting toe touch, side-lying quad). When necessary, movement preparation exercises were performed on the left and right side. 

### 2.3. Height, Body Mass, Body Mass Index (BMI), Body Fat Percentage (BF%)

Height of each firefighter was measured via a stadiometer (Seca North America, Chino Hills, CA, USA) and recorded in inches before being converted to m for this study. Body mass, BMI, and BF% were measured via a foot-to-foot bioelectrical impedance analysis (BIA) machine (Tanita Corporation, Tokyo, Japan). BIA is a practical and cost-effective method for measuring body composition [37,45]. The device used age, height, sex, and activity level to obtain body mass, BMI, and estimate BF%. When conducting the test, firefighters took off their shoes and socks and placed the heels and toes of their feet directly on the scale with forefeet and heels on the electrodes of the device. Body mass was obtained in pounds and converted to kg, while BMI was calculated in kg/m^2^. Based on level of activity, BF% was acquired through both an athletic mode (i.e., firefighter self-reports exercising 3 or more hours per week) or non-athletic mode (i.e., firefighter self-reports exercising less than 3 h per week) [45]. 

### 2.4. Waist Circumference (WC) and Waist-to-Hip Ratio (WHR)

WC provides an indication of body fat distribution [30,31], and the measurement was taken at the narrowest part of the torso between the ribs and iliac crest [38,46] with a tape measure (Sammons Preston USA, Bolingbrook, IL, USA). When conducting the test, firefighters were instructed to remove their shirt, stand with their feet hip/shoulder width apart, arms straight out in front of their body, and maintain a normal breathing pattern. Tension was applied to the tape to fit snugly around the torso and did not indent the skin or compress subcutaneous tissue. The measurement was recorded in cm and at the end of a normal expiration. Hip circumference was also measured in order to derive WHR. The circumference was taken at the largest protrusion of the buttocks [38,46]. When measuring hip circumference, firefighters were instructed to stand with their feet together and arms straight out in front of their body, while squeezing their gluteal muscles. Tension was applied to the tape to fit snugly around the hips and did not indent the skin or compress the subcutaneous tissue. The measurement was recorded in cm and at the end of a normal expiration. After obtaining the waist and hip circumferences of firefighters, WHR was calculated.

### 2.5. Sit-and-Reach

Sit-and-reach provided a metric for low back and hamstring flexibility [47]. The staff used modified equipment that mimicked the sit-and-reach measurement, so established procedures were adopted [48,49]. The test involved the firefighter sitting with both feet pressed against a footboard and knees strapped to a bench. The firefighter slowly pushed an indicator as far forward as possible with their fingertips, which were positioned so the fingertips pushed the indicator. Sit-and-reach distance was obtained in cm.

### 2.6. Grip Strength

Grip strength was measured via a hand grip dynamometer (Lafayette Instrument, Lafayette, IN, USA). Procedures used for this test were adapted from the literature [37,50,51]. Firefighters were instructed to stand with their feet approximately shoulder width apart, grip the dynamometer with the elbow joint at 90°, and while keeping their arms by their sides, squeeze with a single maximal effort. The grip handle was set to the middle phalanx of the middle finger positioned straight across the front of the handle [50]. Firefighters were given two alternating trials for each hand, with at least 30 s of rest between trials. The best score for each hand was recorded in kg and summed to provide a combined grip strength score.

### 2.7. Leg Press

A leg press machine (Cybex, Owatonna, MN, USA) was used to perform a repetition-maximum (RM) test to measure lower-body strength [52,53]. If firefighters had no health risks or injuries and were currently resistance training, the option of a 1 RM test was offered by the staff. If the firefighter declined completing a 1 RM for any reason (e.g., fear of injury), then a 2–10 RM test was attempted to predict a 1 RM. If firefighters were not consistently physically active outside of work, but had no injuries, then a 2–10 RM test was attempted. If some health or injury risk was present, a submaximal baseline measurement was determined only when safe to do so. If significant risk and/or injury was present, the test was omitted. While it would be beneficial to have every firefighter complete a maximal strength test in the same manner, this is not realistic given the population and the need to not injure any individual such that they would miss work [22,24]. A modified rating of perceived exertion (RPE) that featured a 1–10 scale was used after each set to track intensity and to monitor safety.

Standard procedures that followed maximal strength test recommendations were used by staff to administer the leg press test [54,55]. Set 1 was a warm-up set of 6–8 repetitions at 40–60% of the estimated maximum effort (RPE of 4–6), at a starting point of ~81.65–122.47 kg. If the starting weight was too heavy for the firefighter, the weight was reduced until an appropriate weight was reached. Firefighters were instructed to grip the handles on the sides near the safety-stop levers, lower the weight to an approximate 90° angle at the knee joint, then extend the knees without locking to lift the weight, and provide an RPE at the end of the set. After set 1, 60 s of rest was provided. Set 2 was a warm-up set of 2–4 repetitions at 60–80% of the estimated maximum effort (RPE of 6–8), at a weight of ~122.47–204.12 kg, which was followed by a 120-s rest period. Set 3 (and beyond when applicable) involved the firefighter attempting to perform at least 1–2 repetitions, starting at a weight of ~163.29–285.76 kg. If more weight could be lifted after 2 repetitions, the set was stopped, and weight was increased by ~20.41–81.65 kg. After each set, RPE was obtained and a 120-s rest period was given between attempts. If a 1 RM test was declined, repetition maximum attempts continued until firefighters believed the weight was enough. When adequate weight was found, firefighters were then instructed to complete as many repetitions as possible completing between 2–10 repetitions. After the final set was completed, the RPE was recorded along with the total amount of weight pressed in pounds and total number of repetitions. The weight and repetitions were used to predict firefighter’s 1 RM for the leg press [55], which was then converted from pounds to kg. 

### 2.8. Abdominal Crunches

The 90-s crunch test provided a measure of abdominal muscular endurance [56]. This test was adopted as it encouraged greater abdominal muscle activation and limited the contribution of the hip flexors relative to the sit-up [57,58], while also requiring very little equipment [41]. The 90-s crunch test was performed with standard actions [59], and the equipment included a gym mat, stopwatch, and a wall. Firefighters began in the supine position with their knees flexed to approximately 45° and 30.5–45.7 cm from the buttocks. The toes were placed against the wall with both arms across the chest, and both hands grasping opposite elbows to make a box. Staff held a clipboard 15.2 cm from the elbows, instructing firefighters to touch their forearms or elbows to the clipboard when crunching up. On the way down, the lower and upper back and shoulders were to touch the mat. While keeping their hips on the floor, firefighters completed as many crunches as possible in 90 s. Although the test was continuously timed, rest could be taken for up to 5 s at a time. The test was terminated if firefighters reached fatigue, took longer than 5 s of rest after more than one warning, or repeatedly lost proper technique. The number of successfully completed crunches was recorded. 

### 2.9. Push-ups

The 2 min push-up test was utilized to measure upper-body muscular endurance [60,61]. The equipment included a cup that was 12.7 cm tall, a metronome set at a speed of 80 beats per minute (Matrix Electronic Technology Co., LTD, Xi’an, Shaanxi, China), and a stopwatch. Firefighters started in the standard up position [34,37,38,50], with hands shoulder width apart, back straight, head in the neutral position, and the cup beneath the chin. Push-ups were performed to the cadence of the metronome [34], with one beat down and one beat up (i.e., 2 s per repetition). One complete repetition involved lowering the body to the floor until the chin touched the cup, followed by full extension of the arms. The test was stopped when 80 push-ups were completed, 3 consecutive incorrect push-ups were performed, a continuous push-up motion was not maintained with the metronome after 2 warnings, or fatigue. The number of successful repetitions was recorded.

### 2.10. Estimated Maximal Aerobic Capacity (V̇O_2max_)

The Bruce Protocol is a 7-stage graded exercise treadmill test that measures the ability to perform moderately strenuous exercise over an extended time and is used to estimate maximal aerobic capacity [62]. This protocol has been used within the health and wellness program for many years to produce historical normative data and has also been adopted before for aerobic testing in firefighters [63,64,65,66]. Standard procedures were followed for this test [62,63,64,65,66]. Following a 1 min warm-up on the treadmill at a speed of 2.7 km per hour (km/h) with a 0% grade, the Bruce Protocol was initiated, prompting firefighters to begin stage 1 at a speed of 2.7 km/h with a 10% grade. Every 3 min there was an increase in treadmill speed and percent grade, signifying a stage change (Table 1). An RPE scale of 1–10 was utilized at the start of the protocol and prior to each change in stage to monitor the exertion and safety of firefighters. The test was completed when firefighters indicated to staff they would like to stop, or upon staff judgement that a firefighter should no longer continue. Upon completion, firefighters provided a final RPE value and began a 5 min cool-down. The cool-down was broken down into approximately 3 min of walking at a speed of 2.7 km/h with a 0% grade, followed by 2 min of sitting. Overall treadmill times (not including warm-up or cool-down) were recorded by staff and used to estimate V̇O_2max_ (Table 2) [63].

### 2.11. Statistical Analysis

Statistical analyses were processed using the Statistics Package for Social Sciences (Version 28; IBM Corporation, Armonk, NY, USA). The data analysis for this study was based on that from previous tactical research [22,42,67]. Descriptive statistics (mean ± standard deviation [SD]) were calculated for each variable, relative to the combined sample and the sexes. Due to the disparity in sample size between the male and female firefighters, differences between the sexes were investigated by the Mann–Whitney U-test, with significance set at *p* < 0.05. Similar to previous research in first responder populations [37,38,39,60], partial correlations controlling for sex were then used to derive relationships between the body composition (BMI, BF%, WC, and WHR) and fitness (sit-and-reach, combined grip strength, 1 RM leg press, abdominal crunches, push-ups, and estimated V̇O_2max_) tests. Significance was set as *p* < 0.05. Correlation (*r*) strength was delimited as: ±0–0.3 = small; ±0.31–0.49 = moderate; ±0.5–0.69 = large; ±0.7–0.89 = very large; and ±0.9–1 = near perfect [68].

Individual data were then compared to categorical normative data, relative to sex and age. BMI, CVD risk relative to BMI and WC, sit-and-reach, grip strength, push-ups, estimated 1 RM leg press, and estimated V̇O_2max_ were compared to normative data shown by Riebe et al. [40]. BF%, CVD risk relative to WHR, and crunches were compared to normative data presented by Ryan and Cramer [41]. The variables were then profiled using Microsoft Excel (Microsoft Corporation^TM^, Redmond, WA, USA). As the variables each had specific categories, separate graphs were produced for each variable to ensure greater presentation clarity. Male and female firefighters were grouped together according to the respective categories for the different measures, as sex-specific standards were provided where appropriate [40,41]. 

## 3. Results

Subject details, body composition, and fitness testing data for all firefighters combined, men, and women can be observed in Table 3. There were no significant between-sex differences in age. Male firefighters were significantly taller, heavier, had a greater BMI, and greater WC and WHR compared to the female firefighters. The male firefighters were also superior in combined grip strength and the 1 RM leg press. Female firefighters had a greater BF% and sit-and-reach when compared to males. There were no significant differences between the sexes in crunches, push-ups, or estimated V̇O_2max_.

The partial correlation data (controlling for sex) is shown in Table 4. All significant relationships indicated that a better body composition profile (i.e., lower BMI, BF%, WC, and WHR) related to better performance on a fitness test (i.e., further sit-and-reach, greater grip strength and 1 RM leg press, more crunch and push-up repetitions, higher estimated V̇O_2max_). BMI had significant negative relationships with sit-and-reach, 1 RM leg press, crunches (all small), push-ups (moderate), and estimated V̇O_2max_ (large). WC demonstrated significant negative relationships with the same fitness tests as BMI; the sit-and-reach correlation was small, 1 RM leg press, crunches, and push-ups were moderate, and estimated V̇O_2max_ was large. BF% and WHR had significant negative relationships with all fitness tests. There were small correlations with sit-and-reach and grip strength and small-to-moderate relationships with 1 RM leg press, crunches, and push-ups. BF% had a large correlation with estimated V̇O_2max_, while WHR had a moderate correlation.

**Figure 1:**
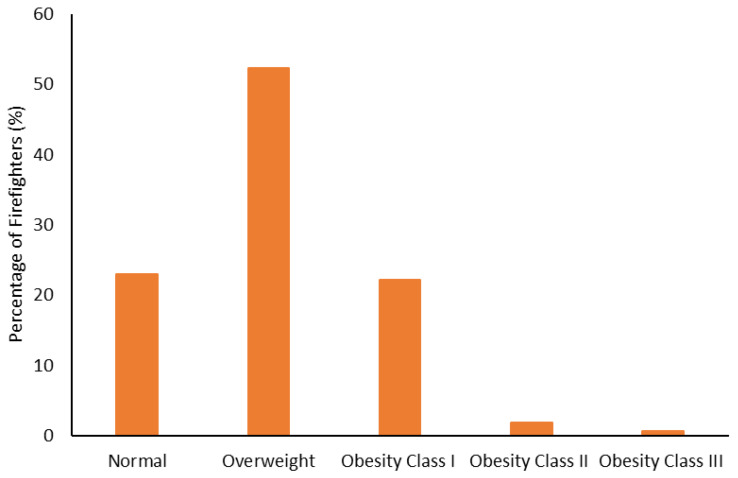
Percentage of firefighters classified according to their Body Mass Index (BMI).

**Figure 2:**
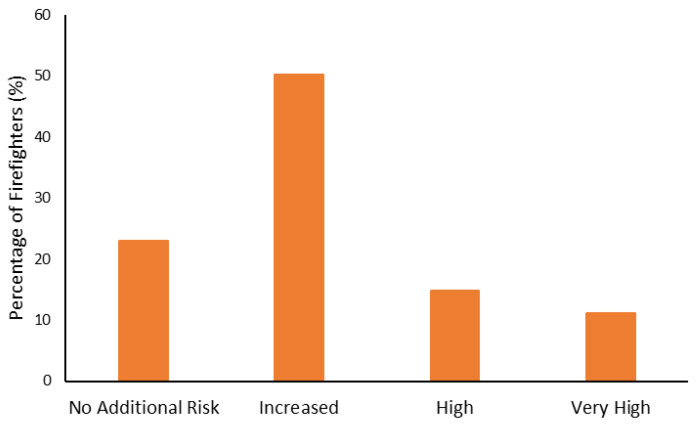
Percentage of firefighters classified according to cardiovascular disease risk relative to waist circumference and body mass index.

**Figure 3:**
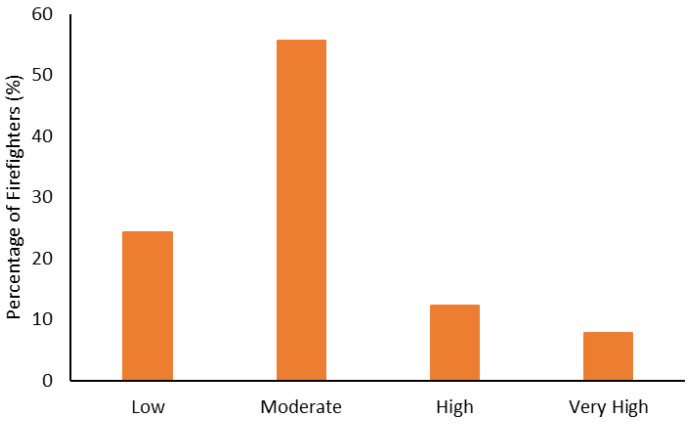
Percentage of firefighters classified according to cardiovascular disease relative to waist-to-hip ratio.

**Figure 4:**
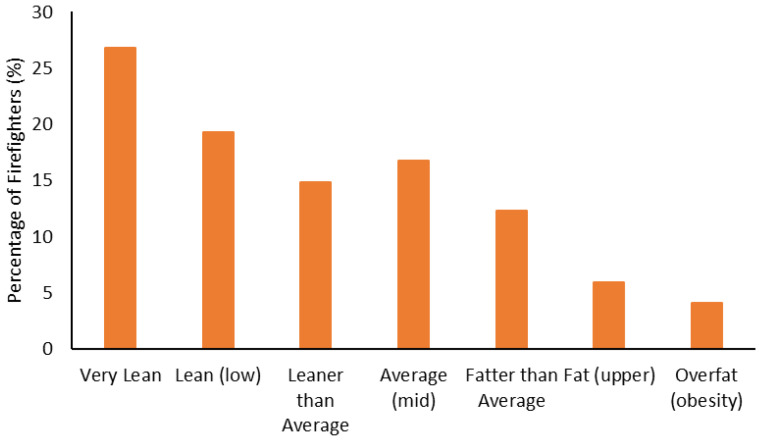
Percentage of firefighters classified according to body fat percentage.

The body composition categorization data are shown in Figure 1, Figure 2, Figure 3 and Figure 4. With regard to BMI, approximately 23% of the firefighters had a normal BMI; just over half were overweight, and almost 25% were Obesity Class I-III. When considering BMI and WC relative to CVD risk, almost 76% of firefighters had an increased risk (or greater) of CVD (Figure 2). However, most firefighters had low-to-moderate CVD risk according to WHR (80%), with 8% classified as high risk (Figure 3). Approximately 61% of the sample were very lean-to-leaner than average when considering BF%, while 22% were fatter than average-to-overfat (Figure 4).

**Figure 5:**
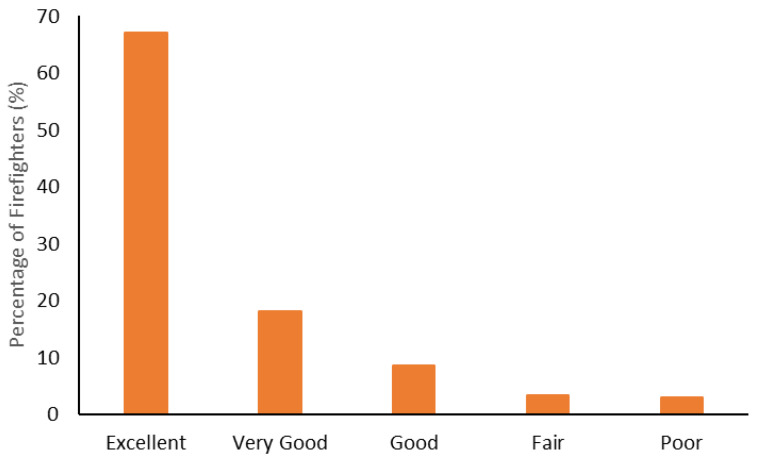
Percentage of firefighters classified according to sit-and-reach.

**Figure 6:**
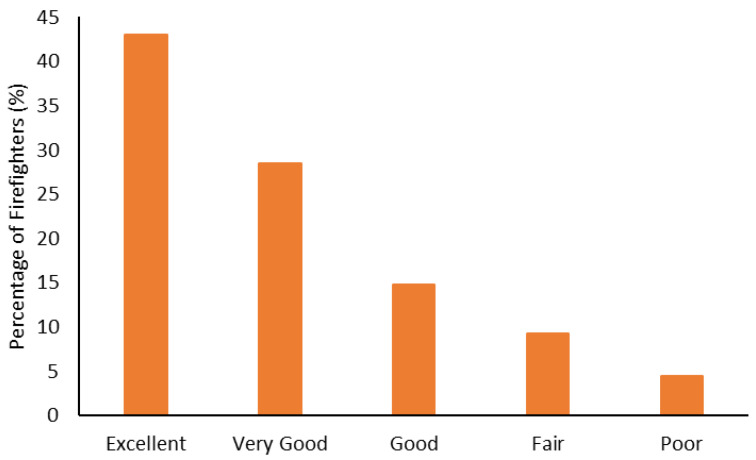
Percentage of firefighters classified according to combined grip strength.

**Figure 7:**
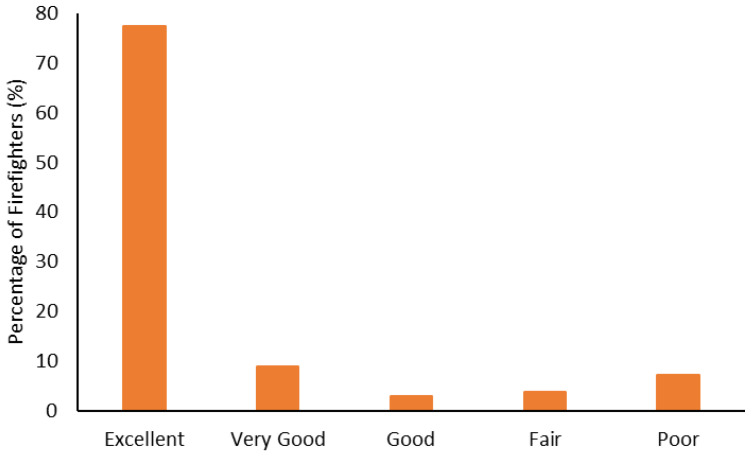
Percentage of firefighters classified according to push-up repetitions.

**Figure 8:**
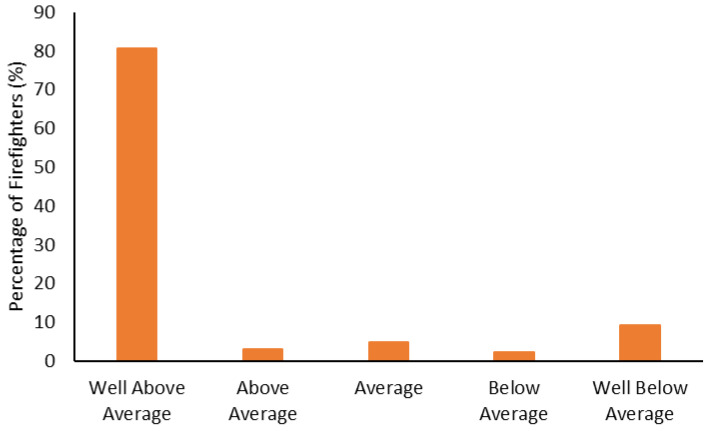
Percentage of firefighters classified according to abdominal crunch repetitions.

**Figure 9:**
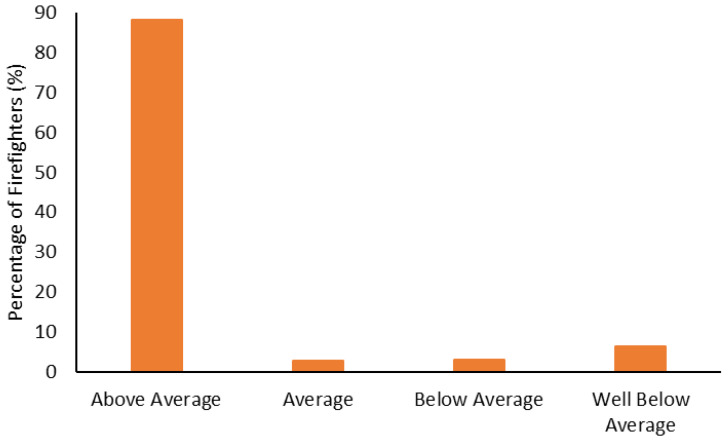
Percentage of firefighters classified according to relative one-repetition maximum leg press.

**Figure 10:**
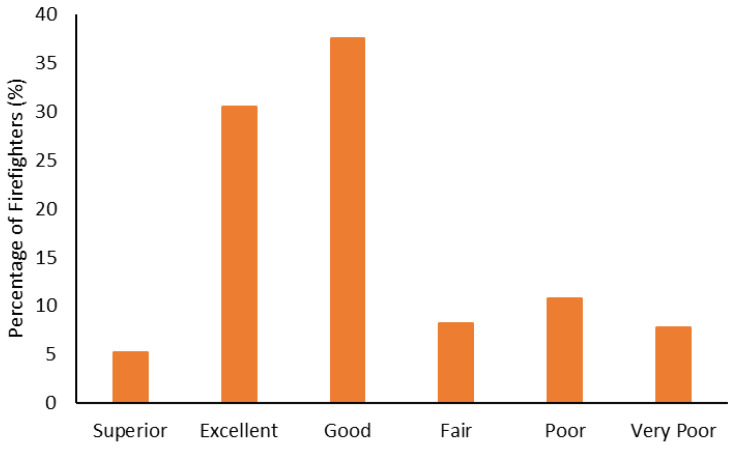
Percentage of firefighters classified according to estimated maximal aerobic capacity.

The fitness categorization data is shown from Figure 5, Figure 6, Figure 7, Figure 8, Figure 9 and Figure 10. Most firefighters were classified as very good-to-excellent in the sit-and-reach (85%; Figure 5), grip strength (72%; Figure 6), and push-ups (86%; Figure 7); well above average in crunches (81%; Figure 8); and above average in their relative 1 RM leg press (88%; Figure 9). There were still 3–7% of firefighters classified as poor for the sit-and-reach, grip strength, and push-ups, while 6–9% were well below average in crunches and the leg press. Most firefighters (73%) had good-to-superior V̇O_2max_; 19% were poor-to-very poor (Figure 10). 

## 4. Discussion

This study provided a correlation and descriptive analysis of the health and fitness of firefighters who were participating in a health and wellness program from one fire department. Firstly, the sexes were compared to provide an initial profile of the firefighters. Although the sample of female firefighters was small in this study (*n* = 12), their data is important to document as many departments are actively trying to recruit more women [35]. Male firefighters were, on average, taller and heavier than female firefighters, which was expected [69]. Some of the between sex differences were typical of previous research in first responders (e.g., the men being superior in the strength tests [grip strength, leg press], the women being superior in the flexibility test [sit-and-reach]) [49,67,70,71]. Interestingly, there were no significant differences between the sexes in crunches, push-ups, and estimated V̇O_2max_. Muscular endurance and aerobic fitness underpins many job tasks required by firefighters [10,11,12], so this was a notable result for both sexes. It should be noted, however, that a minimum V̇O_2max_ of 45 mL/kg/min has been recommended for firefighter personnel [8]. Both male and female firefighters had a mean estimated V̇O_2max_ below this value. This highlights the importance of health and wellness programs, as staff could provide interventions to assist with ensuring fitness of personnel is commensurate with the needed job tasks. Moreover, health and wellness interventions may be needed when considering the body composition and CVD risk of firefighters in this sample.

Analyzing the body composition and relationships to fitness tests in firefighters could provide useful information given the links between obesity and CVD risk [15,16,30,31]. Almost all correlations between the body composition measures and fitness tests from this study were significant. The strongest relationships occurred with estimated V̇O_2max_, which is not surprising given the links between aerobic fitness and favorable body composition [39,72,73]. Nonetheless, it should be noted that except for the correlations with estimated V̇O_2max_, the strength of most of the relationships were small-to-moderate, which is similar to other first responder studies [37,38,39]. Lockie et al. [38] found that from a range of fitness tests (grip strength, push-ups, sit-ups, vertical jump, medicine ball throw, 75-yard pursuit run, arm ergometer, and multistage fitness test), the vertical jump had the strongest relationships to WC and WHR in law enforcement recruits (r = −0.326 and −0.144, respectively). In a study that used the same fitness tests, Collins et al. [39] found the strength of correlations with BF% measured via bioelectrical impedance analysis ranged from ±0.056–0.553 in law enforcement recruits. These data, and that from the current study, would suggest that there are factors other than just fitness that could contribute to favorable body composition (and by extension reduced CVD risk) in firefighters. Improving fitness alone may not be sufficient to reduce body fat and CVD risk in firefighters, which implies other strategies (e.g., nutritional interventions, stress education) should be part of health and wellness programs for firefighters. 

This supposition has support in research conducted on police officers participating in a health and wellness program, where Lockie et al. [22] suggested that health and fitness were relatively disparate qualities. In this study there were very few significant relationships between blood lipids (cholesterol, low-density lipoproteins, high-density lipoproteins, and triglycerides) and multiple fitness measures (sit-and-reach, vertical jump, grip strength, bench press, push-ups, sit-ups, and estimated V̇O_2max_) [22]. On the basis of these results, Lockie et al. [22] suggested that CVD risk should be considered relatively independent from fitness in police officers. Accordingly, it is important to analyze individual officers to ascertain their health and fitness profiles. This will demonstrate any potential limitations in body composition, health, and fitness of the firefighters and provide future directions for health and wellness programs. 

Approximately 77% of the firefighters in this sample were classified as overweight or above according to their BMI (Figure 1). Granted, there are limitations with using BMI to assess health risks and extrapolating body fat in an individual [74]. Indeed, only about 22% of the firefighters were considered fatter than average to overfat according to their BF% (Figure 4). Nonetheless, when BMI was considered with WC, 77% of the sample had an increased risk or greater of CVD (Figure 2). Further to this, approximately 76% of the firefighters had moderate to very high risk of CVD according to WHR (Figure 3). It should be acknowledged that the body composition measures drawn from the archival data in this study did not include lean body mass. Nonetheless, the body composition metrics from the health and wellness program provide practical and established methods for extrapolating CVD risk [40,41], which provides essential information for firefighters given the prevalence of CVD in this population [13,14,15,16]. The current results do help support the need for health and wellness programs in firefighters specifically. These types of programs can provide interventions that target risk factors associated with CVD, notwithstanding other issues that influence the health and well-being of firefighters [22,23,24,25].

Most firefighters had greater fitness compared to the general population. Approximately 94% were rated as good-to-excellent in the sit-and-reach (Figure 5); 86% had good-to-excellent grip strength (Figure 6); 89% were good-to-excellent in push-ups (Figure 7); 88% were average-to-well above average in crunches (Figure 8); 91% were average-to-above average in relative 1 RM leg press (Figure 9); and 73% had good-to-superior V̇O_2max_ (Figure 10). Given the importance of flexibility, muscular endurance and strength, and aerobic capacity in many firefighting job tasks [10,11,12], this is a positive result for these firefighters. It should be noted that the results from this study could be influenced by the healthy worker effect [75,76]. Lockie et al. [22] noted this epidemiological bias in research investigating health and wellness programs for police officers. For the current research, it is possible that fitter firefighters were more likely to participate in the health and wellness program. This could provide the impression that the fire department is fitter than they might otherwise be [22], as the less fit firefighters are not involved with the program. In addition to this, it cannot be surmised from the current data whether the health and wellness program led to better fitness for the firefighters, or that the firefighters who were already fit participated because the program was incentivized. Nevertheless, fire department staff should view the current data as a positive relative to the general fitness of their participating firefighters and note the potential value of health and wellness programs.

Even though the firefighters had aerobic capacity that was generally superior relative to normative standards, the mean of the sample (43.63 ± 8.78 mL/kg/min) was still slightly below the recommended 45 mL/kg/min threshold [8]. These data could be influenced by older firefighters participating in the health and wellness program, as their job responsibilities may have changed over the course of their careers (i.e., more supervisory roles, less physical activity) [21]. Further to this, there were firefighters who were classified as poor or below average in all of the fitness tests (Figure 5, Figure 6, Figure 7, Figure 8, Figure 9 and Figure 10). As multiple fitness domains can assist with job performance [10,11,12], and reduce CVD [14,16] and musculoskeletal injury [77,78] risk, this is less than ideal for these individuals. However, it is a positive that these firefighters were participating in the health and wellness program. Health and wellness program staff would be positioned to provide interventions or education as needed to these firefighters [22]. Future research should track these firefighters over time to see whether their health outcomes improve with program participation.

What was striking was that although many participating firefighters had superior fitness in multiple domains relative to the general population, there was still a large percentage of the sample that had high risk of CVD relative to BMI, WC, and WHR. Despite potential limitations of these measurements, and how some will not consider the muscularity of the individual [74], these are still pertinent results. As noted previously, Lockie et al. [22] suggested that health and fitness were disparate qualities in police officers, demonstrated by limited relationships between blood lipids, which can indicate CVD risk [79,80,81], and multiple measures of fitness. Similar results could be inferred for firefighters in the current investigation (i.e., they were fit but not healthy relative to their risk of CVD). Even with the significant relationships between body composition and fitness shown in this study, the strength of the correlations suggested there was variance in body composition not explained purely by the fitness of the firefighters. In support of Lockie et al. [22], the current results emphasize the need for a multifaceted approach (e.g., health and fitness testing, strength and conditioning programs, dietary interventions, etc.) for health and wellness programs in firefighters to reduce CVD risk and enhance fitness. Furthermore, these results highlight the need for correct outcome measures to be used in physical assessment frameworks as health and fitness are not mutually inclusive [82].

There are study limitations that need to be noted. The health and wellness program involved voluntary and incentivized participation, with approximately 68% of department personnel completing the testing. Further, the current results could be related to the healthy worker effect [22,75,76]. Nevertheless, the focus of the study was on firefighters participating within a health and wellness program, which the all the firefighters in this study were. Firefighters can have different job responsibilities relative to their position in a fire department [21], and also different roles on the fire ground [83], and this could influence their fitness. Overall job responsibilities and fire ground positional roles were not considered in this study. There are limitations with using foot-to-foot BIA to measure BF% [84]. Nonetheless, this equipment is more practical and likely to be used by fire departments than other more expensive and time-consuming equipment [37]. Lean body mass was not included in the body composition methods for this study and should feature in future research. Maximal strength as measured by the leg press was estimated for firefighters in this study, although there were safety considerations associated with this metric [22,24]. This study was cross-sectional, and more research is needed to document whether health and wellness programs can improve a firefighter’s body composition, health profiles, and fitness over the long term. Specific age groups were not analyzed within this study. Future research should investigate specific age ranges of firefighters participating in a health and wellness program (cross-sectional and longitudinal) to document health and fitness differences or changes with age. 

## 5. Conclusions

Male firefighters, on average, had a greater WC, WHR, and were superior in grip strength and the leg press. Female firefighters, on average, had greater BF% and a better sit-and-reach. There were no significant between-sex differences in crunches, push-ups, and estimated V̇O_2max_. Significant relationships between body composition and fitness were documented; however, the strength of the correlations suggested there was variance that could not be explained by fitness alone. Relative to normative data, most firefighters participating in the health and wellness program exhibited better fitness relative to the general population, which could be considered a positive outcome that provided support for health and wellness programs in fire departments. Notably, however, was that while the majority of firefighters appeared to be physically fit, there was a high percentage of the sample that were at an increased risk of CVD relative to their BMI, WC, and WHR. These results highlight the importance of reducing CVD risk in firefighters, especially through enhanced body composition (i.e., reduction of BMI, WC, and WHR). This could be serviced via a multifaceted intervention approach within a health and wellness program (e.g., health and fitness testing, strength and conditioning/exercise programs, dietary interventions, etc.).

## Figures and Tables

**Table 1:** Stages for the Bruce treadmill protocol.

Stage	Time (min)	Speed (km/h)	Grade (%)
1	0–3	2.7	10%
2	3–6	4.0	12%
3	6–9	5.5	14%
4	9–12	6.8	16%
5	12–15	8.0	18%
6	15–18	8.9	20%
7	18–21	9.7	22%

**Table 2:** Estimated maximal aerobic capacity (V̇O_2max_) based on total treadmill time from the Bruce protocol.

Treadmill Time (min)	V̇O_2max_ (mL/kg/min)
5	17.7
6	20.2
7	23.1
8	26.5
9	30.2
10	34.1
11	38.2
12	42.5
13	46.7
14	51.0
15	55.1
16	59.0
17	62.7
18	66.1
19	69.1
20	71.6
21	73.6

**Table 3:** Descriptive data (mean ± SD) for age, height, and body mass; body composition (body mass index, body fat percentage, waist circumference, and waist-to-hip ratio) and fitness test performance (sit-and-reach, combined grip strength, one-repetition maximum [1 RM] leg press, crunches, push-ups, and estimated maximal aerobic capacity [V̇O_2max_]) for male and female firefighters and both groups combined.

Tests	Combined (*N* = 270)	Males (*n* = 258)	Females (*n* = 12)	*p*
Age (years)	42.27 ± 9.63	42.45 ± 9.51	38.42 ± 11.72	0.135
Height (m)	1.80 ± 0.07	1.80 ± 0.07	1.73 ± 0.07 *	0.002
Body Mass (kg)	90.08 ± 13.13	90.61 ± 12.97	78.45 ± 11.47 *	0.002
Body Mass Index (kg/m^2^)	27.87 ± 3.51	27.94 ± 3.43	26.24 ± 4.94 *	0.027
Body Fat Percentage (%)	18.79 ± 6.12	18.68 ± 5.97	21.27 ± 8.69 *	0.215
Waist Circumference (cm)	91.70 ± 9.64	92.25 ± 9.34	78.82 ± 7.37 *	<0.001
Waist-to-Hip Ratio	0.90 ± 0.07	0.90 ± 0.06	0.76 ± 0.05 *	<0.001
Sit-and-Reach (cm)	40.12 ± 9.88	39.67 ± 9.79	49.83 ± 6.37 *	<0.001
Combined Grip Strength (kg)	103.89 ± 15.61	104.98 ± 14.91	80.33 ± 11.58 *	<0.001
1 RM Leg Press (kg)	370.39 ± 125.24	375.26 ± 124.99	265.69 ± 77.87 *	<0.001
Crunches (repetitions)	108.36 ± 45.78	107.94 ± 45.82	118.09 ± 45.79	0.108
Push-ups (repetitions)	38.00 ± 16.46	38.23 ± 16.62	32.45 ± 11.11	0.058
Estimated V̇O_2max_ (mL/kg/min)	43.63 ± 8.78	43.74 ± 8.76	41.17 ± 9.25	0.328

* Significantly (*p* < 0.05) different from the male firefighters.

**Table 4:** Partial correlations controlling for sex between body composition (body mass index [BMI], body fat percentage [BF%], waist circumference [WC], and waist-to-hip ratio [WHR]) and fitness test performance (sit-and-reach, combined grip strength one-repetition maximum [1 RM] leg press, crunches, push-ups, and estimated maximal aerobic capacity [V̇O_2max_]) in male and female firefighters (*N* = 270).

		BMI	BF%	WC	WHR
Sit−and−Reach	*r* *p*	−0.143 * 0.021	−0.220 * <0.001	−0.249 * <0.001	−0.196 * 0.001
Grip Strength	*r* *p*	0.075 0.222	−0.177 * 0.004	−0.007 0.911	−0.151 * 0.014
1 RM Leg Press	*r* *p*	−0.219 * <0.001	−0.389 * <0.001	−0.358 * <0.001	−0.305 * <0.001
Crunches	*r* *p*	−0.291 * <0.001	−0.383 * <0.001	−0.369 * <0.001	−0.289 * <0.001
Push−ups	*r* *p*	−0.396 * <0.001	−0.481 * <0.001	−0.503 * <0.001	−0.358 * <0.001
Estimated V̇O_2max_	*r* *p*	−0.521 * <0.001	−0.638 * <0.001	−0.640 * <0.001	−0.423 * <0.001

* Significant (*p* < 0.05) relationships between the two variables.

## Data Availability

Restrictions apply to the availability of these data due to ethical, legal and privacy concerns.
